# Emotions in Engineering Ethics Education: Systematic Review and Ways Forward

**DOI:** 10.1007/s11948-025-00543-2

**Published:** 2025-07-29

**Authors:** Roland Tormey, Alberto Bellocchi, Pia Bøgelund, Johanna Lönngren, Homero Murzi, Madeline Polmear

**Affiliations:** 1 Ecole polytechnique fédérale de Lausanne (EPFL), Lausanne, Switzerland; 2 Queensland University of Technology, Brisbane, Australia; 3 Aalborg University, Aalborg, Denmark; 4 Umeå University, Umeå, Sweden; 5 Virginia Tech, Blacksburg, USA; 6 Vrije Universiteit Brussel (VUB), Ixelles, Belgium

**Keywords:** Emotion, Engineering ethics, Engineering education, Ethical insight, Moral intuition

## Abstract

Emotion is an increasingly important concept in ethics, education, and engineering. It is also important for the intersection of these three domains: engineering ethics education. Despite extensive research being conducted independently in each field, there has yet to be a synthesis across the fields which would identify key themes, concepts or theories in use, and which would identify conceptual spaces for development. That is the goal of this paper. Our extensive systematic review identified 30 publications relevant to engineering, technology, or computer science education that were substantively focused on emotions and ethics. We coded these abductively and analyzed them thematically to identify underlying theoretical frameworks and concepts. Most publications included theorizations of *emotion, ethics,* or *moral reasoning*, and the ways they are related. Three – primarily psychological – theoretical frameworks were widely used: (A) empathy and pro-social action, (B) moral emotions, and (C) emotional intelligence/ emotional regulation. Possible intersections and relationships between these three frameworks were, however, largely unexplored in the included publications. We conclude that (1) researchers can break down conceptual silos by engaging with the relationships between different theories of emotion and ethics, (2) exploring academic emotions – emotions in the process of *learning* engineering ethics – presents considerable opportunities for further development, and (3) there is a need to broaden the theoretical base to supplement the current individualistic focus with more social theories of emotion and ethics.

## Introduction

Since the 1990s an “emotional revolution” (Sutton & Wheatley, 2003, p. 328) in disciplines such as neuroscience, psychology, and sociology has seen emotion radically re-conceptualized in scholarship. While emotion was long considered as important by scholars from Aristotle, through Descartes and Spinosa to Hume Solomon (2008) argues that – with the exception of Hume – this work was characterized by two features. First emotion was traditionally conceptualized as needing to be held in check by reason because it was seen as “more primitive, less intelligent, more bestial, less dependable, and more dangerous than reason” (Solomon, 2008, p.3). Second, and perhaps more fundamentally, reason and emotion were seen as distinct and separate. Barbalet (2001) suggests that this ‘conventional’ view of the reason-emotion relationship continued to dominate thinking in the modern era via the works of Freud and Weber, even if more alternative conceptualization of emotion, building on prior work by Hume, were available in the work of James and Simel. By the 1990s, however, these alternative conceptualizations moved to the center stage of scholarly work (e.g. Damassio, 1994; Goleman, 1995; Mayer & Salovey, 1993), and the idea that emotion was important for reason – including moral reasoning (Nussbaum, 2001, 2013) – became much more widely accepted. This reimagining of emotions impacted research on education, engineering, and ethics.

Indeed, in ethical decision-making scholarship, the last few decades have seen considerable development on the role of emotion. Work on care ethics (Gilligan, 1982; Noddings, 1988), for example, has emphasized a focus on how socio-emotional networks operate to support, ignore or exploit those who need and those who give care. Alongside this, work on empathy and pro-social behavior (e.g. Batson, 2009; Batson et al., 1997; Hoffman, 2008) highlighted the importance of emotion in moral motivations alongside moral reasoning. More recently, social psychologists, (e.g. Haidt, 2003, 2012) and philosophers (Nussbaum, 2001, 2013; Roeser, 2010, 2012) have explored the roles of specific ‘moral’ emotions such as anger, guilt, shame, compassion, and fear in ethical decision-making.

Emotion is not only important in terms of its contribution to ethical decision making but also because of its growing importance for engineering. While engineering is often understood as the production of rational, scientific, and technical solutions to real-world problems (Cech, 2018), Lönngren et al. (2024) have recently highlighted, there has been a rapid expansion in research on emotions in engineering education. In that emerging body of literature, emotions are studied in relation to domains such as engineering design, collaboration on complex problem-solving, as well as in peace and development engineering (Hess et al., 2017).

Emotion also plays an important role in engineering ethics learning. There is evidence, for example, that including emotional content in cases may improve engineering students’ ethical learning (Thiel et al., 2013). Similarly Higgs et al. (2020) found that describing specific emotions of the protagonist in ethics cases increased ethical motivation. Watts et al. conducted a meta-analysis of ethics education in science (Watts et al., 2017). They found that the educational process that had the strongest positive impact on learning in ethics education was ‘emotional analysis’ (ahead of ‘forecasting’ and ‘analysis of consequences’ and its impact was substantially greater than educational processes focused on ‘values’ or ‘meta-ethical’ analysis).

Despite this evidence for the importance of emotion in engineering ethics education, the role of emotion in engineering ethics education has not yet been adequately explored. For example Hess and Fore (2018) systematically reviewed the literature on engineering ethics interventions in the US, and noted that “having students reflect on their own as well as others’ emotions” (2018, p. 575) is an emerging trend, but emotion was not an important variable in their analysis. Similarly, in their review of the field of engineering ethics Barry and Herkert (2014) mentioned emotion only once and only in passing. Lönngren et al. (2021, 2023, 2024) systematically reviewed publications that significantly focus on engineering education and emotion; however, ethics did not emerge as a major theme in this analysis. So, while the intersection between engineering, ethics, and education is evidently a space in which emotion is important, if this field of research and practice is to develop, there is a need to systematically describe the field.

Indeed, a systematic review identifying key themes, concepts, or theories currently in use could identify gaps in research topics, theories, and approaches used, and could critically evaluate the existing base of knowledge. That is the goal of this paper.

## Literature Review

### How are Emotions Conceptualized?

There is not a single agreed-upon definition of ‘emotion’ (Frijda, 2008, p. 68; Bellocchi, 2019), but it is useful at this point to introduce some shared understanding of emotion as well as important points of difference that emerge in literature.

Barbalet (2001) argues that the definition of emotion was contested over the nineteenth and twentieth centuries. The dominant position, which he refers to as the ‘conventional’ view, saw emotion as distinct from and opposed to reason. Building on a Cartesian dualism, he argues, thinkers such as Freud located the emotions as part of the id and a biological function distinct from memory, judgement and reasoning. Emotion was seen, from this perspective, as effectively defined as bodily disruptions of reason. This view was, however, always contested. For example, William James, described rationality as being a ‘sentiment’ (that is, an emotion). “From James’s perspective”, Barbalet writes (Barbalet, 2001, p. 45) “reason and emotion are not opposed phenomena but distinct names for aspects of a continuous process”.

If the ‘conventional’ view of emotion remains dominant in public perception and folk-theories, most contemporary writers tend to see emotion and reason, intellect and body, as more interlinked. Most contemporary understandings of emotions conceptualize them as multicomponent phenomena that influence how one assesses, decides, and acts. The multiple components involved include: appraisal of a situation, which is linked to physiological changes in the person’s body; a subjective experience of those changes; and a tendency to act and think in particular ways (Shuman & Scherer, 2014). Part of this conceptualization of emotion is the idea that emotion and cognition are interlinked, with cognition contributing to appraisals of a situation and emotion contributing to a tendency to think and act in particular ways.

An important point of difference to emerge from the literature is whether emotion is understood as an individual or a social phenomenon. Some researchers frame emotions in primarily individualistic ways, focusing on the ways (1) a person’s appraisals give rise to different emotions (e.g. Pekrun & Linnenbrink-Garcia, 2014), (2) emotions are linked to or impact upon an individual’s judgement (e.g. Lerner et al., 2015), including moral judgement (Nussbaum, 2001, 2013), and (3) an individual can regulate their own emotions (Gross, 1999, 2014). Other researchers frame emotions in more social terms, emphasizing that many of the components of emotions involve socially constructed, cultural definitions and constraints as to how people should behave in particular cultural contexts, and that culture also provides linguistic labels which frame the bodily changes and subjective experience of emotions (Turner & Stets, 2005, p. 9). Such social accounts typically see emotions as linked to power, inequality, identity, and social structures (Ahmed, 2014; Boler, 1999; Zembylas, 2007). This difference between individualistic and social accounts of emotion might be thought to mirror the distinction between micro- (individualistic) and macro- (social) ethics which is regularly cited in the engineering ethics education literature (Herkert, 2005).

### Prior Reviews on Emotion in Education

Research on emotion in educational settings has burgeoned in the last two decades, leading to numerous reviews and syntheses of the topic (Ajjawi et al., 2021; Ba & Hu, 2023; Bellocchi, 2019; Bellocchi & Amat, 2022; Davis & Bellocchi, 2018; Gomez-Garibello & Young, Gomez-Garibello & M, 2018; Henritius et al., 2019; Lei et al., 2021; Olson et al., 2019; Sharp et al., 2016; see also chapters within Pekrun & Linnenbrink-Garcia, 2014; Schutz & Pekrun, 2007; Schutz & Zembylas, 2009). These reviews reflect the distinction drawn above between individualistic and social framing of emotions.

Reviews by Schutz and Pekrun (2007) and Pekrun and Linnenbrink-Garcia (2014), for example, reflect a more individualistic conceptualization of emotion, which relies on the view that emotions are internal mental states alone. They highlight the extent to which emotion is central to both teaching and learning and are important in interaction in classrooms (social emotions), in students’ reactions to specific subject content (topic emotions), in students’ experiences in trying to understand new content (epistemic emotions), and in students’ experiences of success and failure in learning (achievement emotions). A key theoretical framework underpinning much of this research is the control-value theory (Pekrun et al., 2002), which posits that learning is impacted by a learner’s emotions which in turn are linked to their appraisals of having control over their success in learning and their appraisals of the value in the material being learned. This theory has been applied in disciplines such as science and mathematics education, as well as in broader educational topics including reading and writing, and learning technologies (see Pekrun & Linnenbink-Garcia, 2014). Within these individualistic theories of emotion, the social milieu is a source of external stimuli for emotion or provides targets at which one’s emotions are directed. This approach stands in contrast to social theories of emotion.

Davis and Bellocchi (2018) and Bellocchi & Amat (2022) focus on reviews of studies with social theoretical foundations. Such approaches tend to emphasise that learning, including learning to teach, is not simply a process of information exchange but rather a process of identity development which is discursively constructed in communities and filtered through dominant ideologies (Zembylas, 2005). Rather than analysed in terms of individual experiences, social approaches see emotion in classrooms, for example, in terms of interaction rituals in which a group’s shared emotional experiences are seen as playing a key role in the formation of a group as well as a sense of shared purpose and focus (Bellocchi, 2017).

In addition to non-systematic literature reviews, we also identified several systematic reviews on emotion in education (Ajjawi et al., 2021; Ba & Hu, 2023; Gomez-Garibello & Young Gomez-Garibello & M, 2018; Henritius et al., 2019; Lei et al., 2021; Olson et al., 2019; Sharp et al., 2016). A number of these were aggregate reviews (aimed at summing up what is known about a narrowly defined specific topic) rather than configurative reviews (aimed at organising different theories and approaches used in a domain (see Gough et al., 2017). Topics addressed in these systematic reviews included teacher emotion management, medical and healthcare feedback practices, measurement with wearable devices, Asian teachers’ emotions, assessment rater judgements, virtual learning environments, educational games, and assessing student boredom. Alongside these existing reviews on emotion in education, recent years have seen the emergence of several configurative reviews of literature on emotion in engineering education (Lönngren et al., 2021, 2024). These suggest that the pattern of research in engineering education seems somewhat different from that found in the broader emotion in education literature. In many studies of emotion in engineering education, emotion is an emergent theme rather than the primary focus of the research and, as such, emotion is often not coherently conceptualized. Lönngren et al. (2024) also note that in engineering education, emotion is generally explored as a learning outcome rather than as something experienced as part of the process of learning. This focus on emotion as a learning objective saw a heavy focus on the (individualistic) concept of emotional intelligence as well as related concepts such as awareness of one’s own emotions, empathy for emotions in others, and emotion regulation. Emotions in the process of learning were far less frequently cited (other than anxiety, which did have some prominence), while social, critical, and feminist theories of emotion were seldom mentioned and never thoroughly applied in the reviewed publications. Although it is mentioned as a concept which is linked to emotion, engineering ethics education is not substantively treated in these reviews.

In summary, research in other fields of learning (such as science and mathematics) might lead us to expect that in the field of engineering ethics education there would be some focus on emotions associated with learning experiences including a focus on emotions linked to achievement, to topics being studied, and to a sense of understanding or confusion (epistemic emotions). However, we do not see much of this focus on academic emotions in the literature on engineering education, and we have no evidence about the situation in engineering ethics education. We might also have expected that alongside individualistic theorizations we would also see some focus on social theories of identity formation, on emotional interaction rituals, and on questions of power, discourse or ideology in engineering ethics education. Again, we see effectively none of this in the engineering education literature and have no evidence about engineering ethics education in specific.

Although the intersection between engineering, ethics, and education is evidently a space in which emotion is important, if this field of research and practice is to develop, there is a need to systematically describe the field. The goal of this paper is to carry out a configurative systematic review identifying key themes, concepts, and theories currently in use; to identify gaps in research topics, theories, and approaches used; and to critically evaluate the existing base of knowledge.

## Methodology

This review was conducted by an international and interdisciplinary group of researchers. We are based in academic contexts in six countries (Australia, Belgium, Denmark, Sweden, Switzerland, United States), but some of us have cultural backgrounds in additional countries in Europe and Latin America. Our disciplinary backgrounds include sociology, educational sciences (specifically engineering education and science education), and engineering. Several of our team are particularly interested in sociological and critical perspectives on emotion (and this undoubtedly contributed to our surprise at the lack of social and critical perspectives in this literature, and thus to some of our conclusions). The first author led the work, ensured communication with the rest of the group, assigned research tasks to group members, and monitored progress in each step of the project. All members contributed substantially to all phases of the project, and the collaborative exchanges and critical discussions between team members was a significant asset to the project.

**Fig. 1:**
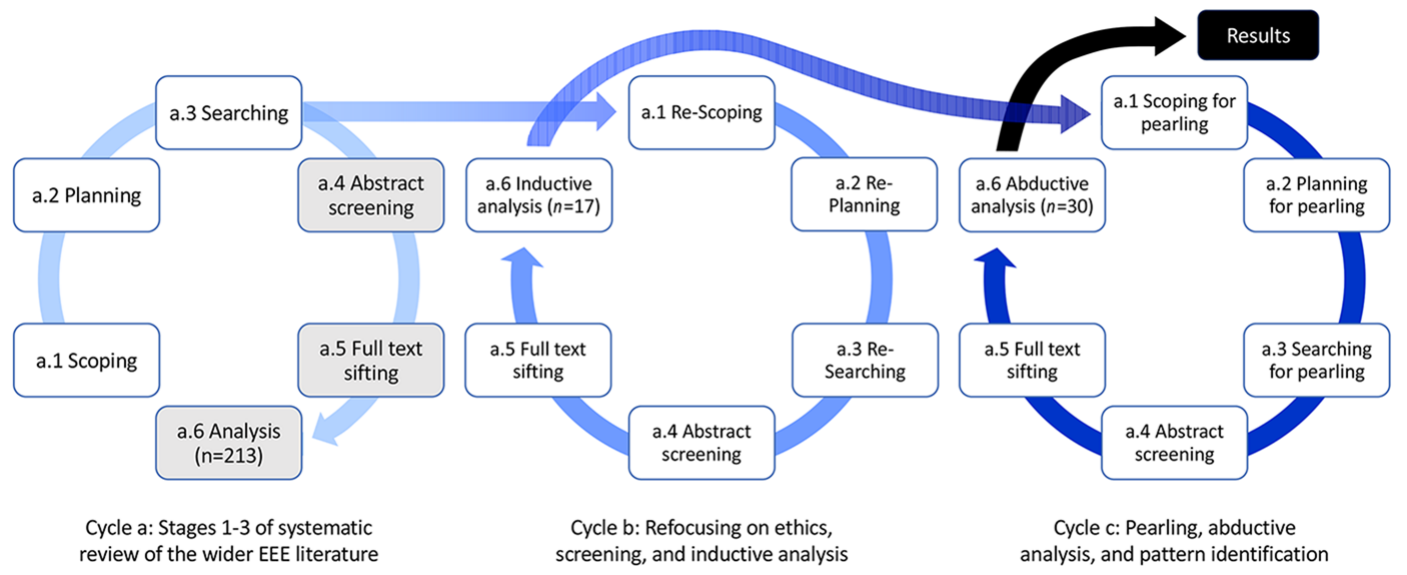
Overview of the three cycles of selection and analysis for the systematic review

The systematic review described in this paper proceeded in three cycles. The first cycle (a) was part of the more extensive systematic review of the ‘Emotions in Engineering Education’ (EEE) literature described in the previous section (Lönngren et al., 2021, 2023, 2024). We scoped, planned, and searched the EEE literature. At this point it became apparent to us that ethics was not a prominent theme in the wider dataset (appearing in less than 10% of publications) and that it would, consequently, not figure prominently in the overall systematic review. Given the importance of emotion in engineering *ethics* we decided to carry out a more focused review on only those papers that substantively referred to ethics. Thus the second cycle (b) narrowed the focus to emotions in engineering *ethics* education. An inductive analysis of the selected publications suggested that there was something meaningful to be said about the field. We therefore proceeded in a third cycle (c) to identify missing publications, complete abductive coding of all publications, and identify patterns across publications (Fig. 1).

In designing the methods for our review, we used an iterative approach inspired by Siddaway et al.’s (Siddaway et al., 2019) description of six stages in conducting systematic reviews: (1) scoping, (2) planning, (3) searching, (4) abstract screening, (5) full-text sifting, and (6) extracting and synthesizing information.

### Cycle A: Sampling the Wider EEE Literature

*Stage a.1: Scoping*. To ensure an inclusive review of the highly fragmented EEE research field, we first applied a broad scope, aiming to capture as many scientific EEE publications as practically feasible.

*Stage a.2: Planning*. To plan the sampling and screening processes for the wider review, and given that the literature included quantitative and qualitative empirical studies as well as conceptual publications, we combined two widely used approaches: PICO (Population, Intervention, Comparison, and Outcomes) and SPIDER (Sample, Phenomenon of Interest, Design, Evaluation, and Research type) (Borrego et al., 2014; Cooke et al., 2012; Methley et al., 2014). Our combined framework focused on *Sample, Setting, Phenomenon of Interest, Outcomes*, and *Publication type*.

**Fig. 2:**
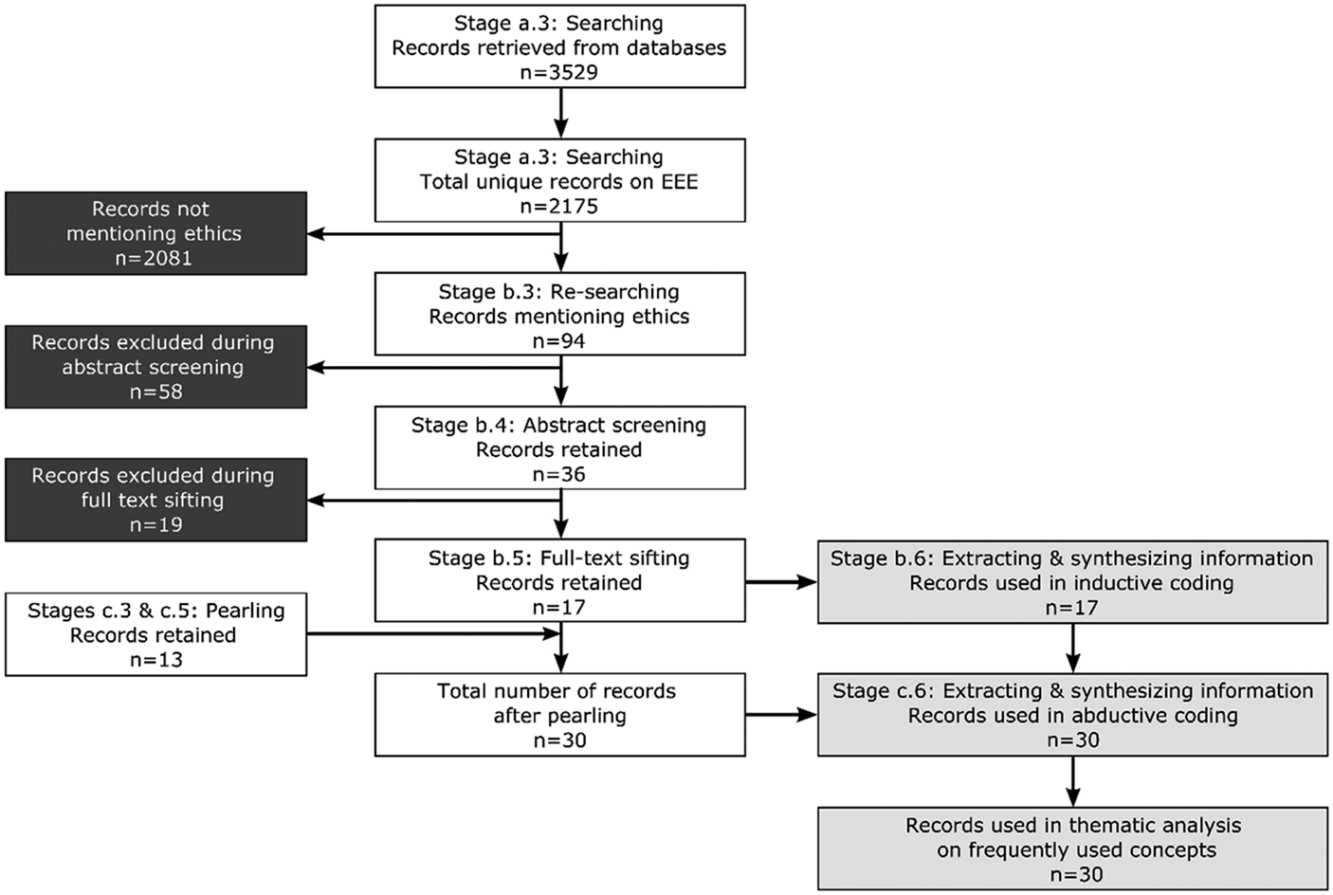
Overview of the number of publications in- and excluded at different stages in the selection and analysis process

*Stage a.3: Searching*. We searched eleven databases, including general databases (Scopus, Web of Science, Academic Search Complete), educational/social science databases (ERIC, IBSS), a psychological database (APA PsycInfo), an engineering database (Engineering Village), and databases specialized on eBooks and theses (eBook Central, Dissertations & Theses Global, Open Thesis). We used the following search string to search title, abstract, and keywords fields in peer reviewed publications: ((emoti* OR affective OR feeling*) AND (“engineer* educat*” OR “technology educat*” Or “engineering stud*” OR “engineering instruct*” OR “engineering facult*”)). This search was conducted in early September 2020, and yielded 3,529 records. Excluding duplicates, we retained 2,175 records (Fig. 2).

At this point in cycle a, the work with the review described in this paper split off into cycle b (Fig. 2), while the remainder of cycle proceeded with the systematic review of the wider field (Lönngren et al., 2021, 2023, 2024).

### Cycle B: Re-Focusing on Ethics in EEE, Screening, and Inductive Analysis

*Stage b.1: Re-scoping*. Since comparatively few publications on ethics were included in the initial dataset, we recognized ethics would not emerge as a major theme in the wider review, and so it was decided to also undertake a more focused review on emotion in engineering ethics education.

*Stage b.2 and b.3: Re-planning* and*: Re-searching*. To do this, we refined our initial search (Stage a.3). We used Zotero reference management software to search for publications mentioning “ethic*” in title or abstract fields (this specificity was necessary to avoid retrieval of articles that report their research ethics protocols rather than focusing on ethics as a topic of research). This search yielded 94 records.

*Stage b.4: Abstract screening*. We developed a detailed abstract screening codebook with the following criteria: (1) *Sample* and/or *Setting* must be related to engineering, technology, and/or computer education; (2) *Phenomenon of interest* and/or *Outcomes* must be related to emotions and ethics; and (3) *Publication type* must be academic. All items were screened independently by two researchers, taking note of the reasons for their decision. In case of disagreement, we combined two common approaches for resolution: analyzing reasons for disagreement (based on the initial screeners’ notes) and involving a third researcher who re-applied the codebook for another round of screening (Petersen & Ali, 2011; Taylor et al., 2021). We excluded 58 items at this stage, leaving 36 items for full-text sifting.

*Stage b.5: Full-text sifting*. To ensure that items in our review were “indeed appropriate for inclusion” (Siddaway et al., 2019, p. 764), we refined the codebook to include only items that were (1) not only related to but also *relevant* for engineering, technology, and/or computer science education; (2) not only related to but *substantively focusing* on emotions and ethics. Again, all items were sifted independently by two researchers, and disagreement was resolved by a third researcher. As a result, 19 items were excluded, leaving 17 for further analysis.

*Stage b.6: Extracting and synthesizing information (inductive analysis)*. Reading through the 17 publications, we first developed individual analytical notes on themes, concepts or theories, research methodologies, and research contexts. Then, comparing our notes, we inductively developed 14 sets of overarching codes, each with a number of sub-codes, for in-depth analysis. For example, we identified a set of codes pertaining to conceptualization of ethics, including the following subcodes: ethical becoming, awareness, decision-making, risk, moral reasoning, moral judgement, ethical theories, and ethical dilemmas, among others (Appendix I).

### Cycle C: Pearling, Abductive Coding, and Pattern Identification

*Stage c.1: Scoping for pearling.* During a citation analysis for the wider systematic review (Stage a.6), we identified several publications in *Science and Engineering Ethics* that were referenced in included publications but had been missed in our initial search. This insight motivated us to conduct pearling to identify other publications that may have been missed in the initial search (Booth, 2016).

*Stages c.2 and c.3: Planning* and *Searching for pearling*. We reviewed all 532 references in the 17 publications identified at Stage b.6. Based on titles and publication outlets, we selected 34 items as potentially relevant for this review.

*Stages c.4 and c.5: Abstract screening* and *Full-text sifting of pearled items*. Due to the small number of pearled items, we combined abstract screening and full-text sifting in this cycle, repeating the process outlined in Stage b.5 for the pearled items. This process resulted in the inclusion of 13 additional publications and a total number of 30 included publications (Appendix II).

*Stage c.6: Extracting and synthesizing information (abductive analysis)*. Starting from the inductively developed codes from Stage b.6, we used Dedoose qualitative software to abductively code all 30 publications. This meant subsequently generating and applying new codes (Vila-Henninger et al., 2022) when we encountered themes, concepts or theories, research methodologies, or research contexts that were not covered by the codes from Stage b.6. Each publication was coded independently by two researchers.

Next, we summarized and discussed the resulting code patterns during a full-day virtual workshop with all authors. Aiming to identify the most prominent themes, concepts, and theories in the reviewed literature, we developed a coding matrix showing which codes were applied to which publications. For each set of codes, we then selected the subcodes that were applied to the largest number of reviewed publications. For each of the selected codes, we then exported all coded excerpts to an excel file and mapped different ways authors used each of the coded concepts in the reviewed publications. Finally, we developed a co-occurrence matrix showing which codes were applied to the same publications, which allowed us to analyzed similarities and differences in how key concepts were used across publications.

## Results

### Overview of the Publications

We begin by providing a description of publication features such as nature of co-authorship, and geographic distribution of principal authors. Two of the publications were co-authored by scholars affiliated with institutions from more than one country, with the remaining 28 authored by scholars affiliated to institutions within one country. A large majority of the authors (25/30) were affiliated to institutions in the United States of America. Other authors were affiliated to institutions in The Netherlands (2 publications), as well as Canada, Finland, Romania, Puerto Rico, or South Africa (1 paper each).

Journal articles accounted for half of the publications included in the review (15/30). Of these, 9 had been published in *Science and Engineering Ethics*. Conference publications accounted for a substantial proportion (11/30); 9 of these had been presented at the American Society for Engineering Education Annual Conference. Thus, only two sources accounted for more than half (18/30) of the publications. Finally, the reviewed publications included three book chapters and one magazine article. Half of the reviewed publications (15/30) did not describe any systematic collection or analysis of empirical data – they were either entirely discursive, described pedagogical practice without systematically evaluating it, or both. Among the publications describing empirical studies, qualitative methods were used in eight publications, quantitative methods in four, and mixed methods in the remaining three.

Having established this basic overview of the corpus of publications, our focus in the following analysis was to identify key themes, concepts, and theories currently in use in the field of emotion in engineering ethics education, and to identify spaces for development within the field.

Several emotion concepts were emergent in the publications in our dataset: the most commonly coded were “compassion/care”, “emotion regulation/management” “emotional awareness/sensitivity”, “emotional intelligence”, “socio-emotive competences”, “emotion words”, “empathy/sympathy/perspective-taking”, and “moral emoting/moral emotions”. For ethics, the most frequently used codes were “ethical theories”, “ethical action/behavior/conduct”, “ethical reasoning/reflection/deliberation”, “moral values/norms/stances”, and “macro-ethics/social responsibility/social implications/socio-scientific issues”.

In line with a thematic analysis approach (Terry et al., 2017) main themes were developed through exploring relationships and cooccurrences between these codes. The goal of this work was to identify a set of core concepts that underpinned a theme and were shared across a range of codes. Three conceptual frameworks linking emotion and ethics emerged as underpinning the theoretical grounding in our data:

- Empathy
- Wider moral emotions and risk judgements
- Emotional intelligence (EI) and emotion regulation

These three frameworks underpinned 27 of the 30 publications in our dataset. In the remaining three publications (Frey, 2010; Hashemian & Loui, 2010; Ramachandran et al., 2015) the link to ethics was not theorised explicitly.

### Empathy

Most of the reviewed publications (27/30) discuss empathy (often conflated with ‘care’ in its treatment). Rather than narrow and specific definitions of empathy, the concept is generally used in omnibus terms as a complex, “holistic,” and “highly nuanced” (Hess et al., 2017, p. 536) concept that involves both cognitive (imagining oneself in the position of another) and emotional (joining the emotional experiences of another) elements (Strobel et al., 2013). Hess and Fila (2016) identify four dimensions of empathy: experiencing distress at another’s distress (an inwardly focused, emotional element), experiencing concern or happiness for another (outwardly focused, emotional element), imagining how one would feel if they were another (inward, cognitive element), and imagining how another thinks or feels (outward, cognitive element). Rather than focus on one specific definition or component of empathy, where the concept of empathy is used in the dataset, it is commonly with reference to this broad-based, multi-dimensional understanding of the term (e.g. Hashemian & Loui, 2010; Hess et al., 2021; Hess & Fila, 2016; Hess et al., 2017; Lappalainen, 2015; Strobel et al., 2013; Walther et al., 2017).

Many of the papers that include a focus on empathy see it as a broader desirable skill or attribute of engineers, rather than one that is only linked to ethics. It is seen as important, for example, in professional relationships (Hess et al., 2017; Lappalainen, 2015; Strobel et al., 2013; Walther et al., 2017); conflict management (Hess et al., 2017; Lappalainen, 2015; Strobel et al., 2013); and job satisfaction, professional development, and career success (Hess et al., 2017; Strobel et al., 2013; Walther et al., 2017). The papers identify several explicit linkages between empathy and ethics. First, empathy is seen as bringing the perspective of those affected by engineering decisions into those decisions (Campbell et al. Campbell, 2013; Gupta et al. Gupta et al., 2015; Hess & Fila, 2016; Hess et al., 2017, 2021; James et al., 2018). When referring to empathetic caring, for example, Campbell et al., (Campbell, 2013, p. 18) note “This conception of empathy encourages the respect and autonomy necessary for limiting paternalistic actions on behalf of [the engineer as] the care-giver”. In this way, empathy can be explicitly linked to an ethics of care (Campbell et al. Campbell, 2013; Hess & Fila, 2016). Interestingly, while empathy is treated alongside care in the data set (Hess et al., 2021; Strobel et al., 2013), it is often not explicitly linked to an ‘ethics of care’ perspective. Empathy is also linked to moral motivation as providing a drive to act on another’s behalf (Han, 2015; Hess et al., 2021; Hess & Fila, 2016; Hess et al., 2017; Kim & Jesiek, 2019; Lappalainen, 2015; Strobel et al., 2013; Thiel et al., 2011; Walther et al., 2017). More broadly, empathy was also seen as important in contributing to positive organizational values (Guntzburger et al., 2019; Gupta et al. Gupta et al., 2015; Hess et al., 2017; James et al., 2018; Lappalainen, 2015; Roeser, 2012; Strobel et al., 2013; Walther et al., 2017).

Since the role of empathy in ensuring stakeholder perspectives in the design process was seen as a capacity integral to engineering itself, empathy was sometimes framed as an important strategy for bringing ethics into the heart of engineering practice. For example, authors describe empathy as a professional way of being for engineering (Strobel et al., 2013; Walther et al., 2017). Strobel et al. (Strobel et al., 2013, p. 147) present data arguing that “engineering … is intrinsically empathetic and/or caring”. While the value of empathy is largely uncontested in the publications in our dataset, some publications acknowledge that this view is not widely shared in engineering more generally; Strobel et al. (2013) find that engineers expect their colleagues to not care about, or even be familiar with, empathy and care. Similarly, Campbell et al. (Campbell, 2013, p. 111) describe empathy and care as a “missing dimension in engineering.”

Consistent with this focus on the value of empathy in engineering practice, most of the dataset addresses empathy as a *learning goal* (rather than as an academic emotion experienced in the *learning process*). Empathy is often described as a learnable skill or competence (Campbell et al Campbell, 2013; Hess et al., 2017; James et al., 2018; Lang et al., 2018; Lappalainen, 2015; Roeser, 2012; Strobel et al., 2013; Walther et al., 2017). Authors also describe empathy in terms of moral development and “empathic formation” (Hess et al., 2021, 2017). For example, students are expected to develop more empathic dispositions or ways of being (James et al., 2018; Lang et al., 2018; Strobel et al., 2013), and feeling for others (Campbell et al Campbell, 2013; Hess et al., 2021, 2017; Lee et al., 2017; Strobel et al., 2013; Walther et al., 2017).

We found very few discussions on empathy as part of a learning process. Some authors briefly reflect on methods for teaching empathy (e.g., through teamwork and stakeholder involvement [Strobel et al., 2013]) and on how it can influence learning. Regarding the latter, empathy is described as an untapped learning opportunity (Hess et al., 2021) that can increase students’ motivation to learn (Strobel et al., 2013); trigger cognitive dissonance (Hess et al., 2021); and facilitate case-based learning (Thiel et al., 2011). However, some authors also argue that empathy can hinder learning if it is not appropriately moderated through emotion regulation (we will return to this below) (Hess et al., 2021; Walther et al., 2017). For example, some authors argue that empathy can trigger overwhelming distress (Berne, 2018; Hess et al., 2021).

In addition to these conceptualizations, empathy is also referenced in work on moral emotions more broadly. This is addressed in the next section.

### Wider Moral Emotions

While empathy (including emotions of compassion and distress-at-another’s distress) has long been identified as playing a role in pro-social behavior (Hoffmann Hoffman, 2008), the last two decades have seen a shift to a focus on other emotions such as guilt, anger, shame, and awe which are also thought to contribute to ethical behavior. These are referred to as *moral* emotions (Haidt, 2003). Two perspectives on such emotions are evident in the dataset: the ‘ethical insight’ and the ‘ethical intuition’ perspectives (Lee et al., 2017).

First, emotions can be seen as providing information which allows for a richer and more complete moral judgement than would be provided by reason alone. In our dataset, Roeser, for example, argues that moral emotions play a role in good ethical judgement in that they provide the engineer with information about risk which would not be available from rational means alone: “emotions are a necessary source of moral reflection about risky technologies, this means that the emotions of engineers should play a role in the design of risky technologies” (Roeser, 2012 p. 108). Emotions here are not envisaged as replacing ethical reflection and moral reasoning, rather they are perceived as providing an ‘ethical insight’ which would not be available to reason alone. In our dataset twelve papers refer to moral emotions other than empathy. Of these, five (Guntzburger et al., 2019; Kastenberg, 2014; Lee et al., 2017; Roeser, 2012; Sunderland et al., 2013) made substantial reference to an ‘ethical insight’ perspective, typically based on the work of the philosopher Sabine Roeser (including one paper by Roeser herself).

Another way of viewing moral emotions is that the emotion is itself a rapid and implicit judgement (an intuition) as to right and wrong. According to this view, the role of rationality is secondary: reason provides post-hoc justifications for moral judgements already made intuitively. Four publications in our set (Gelfand, 2016; Han, 2015; Kim & Jesiek, 2019; Lee et al., 2017) made substantial reference to an ‘ethical intuitionist’ perspective (typically with reference to the work of social psychologist Jonathan Haidt, 2001). Gelfand, for instance, cites Haidt as arguing that emotions are not information for judgement but rather are judgements themselves (Gelfand, 2016). He argues that there is, therefore, a need for students to understand the way psychological processes affect their judgement in order to avoid students simply being led by their own intuitions. Interestingly while Roeser and Haidt both focus on moral emotions, there was little overlap in our dataset between papers citing these two authors. Publications typically argued for one perspective or the other rather exploring or testing both. Only one publication (Lee et al., 2017) substantially engaged with both ethical insight and intuitionist perspectives, and sought to test in which circumstances engineering students rely on reason or intuition when making moral judgements.

Several studies referred to moral emotions that emerge in ethics *learning*. James et al. (2018) found that a student describing role plays in a design activity used the term “violated” and described feeling angry that, in their case study, the regulatory body that was supposed to guarantee clean water was responsible for water pollution. Hashemian and Loui (2010) found that the case they presented gave rise to students feeling scared, annoyed, and “bad,” and students who proposed a passive response to a given case reported feeling guilt. Thiel et al. (2011) specifically manipulated the intensity of moral emotions felt by the case study protagonist and found that in some cases this had a positive impact on participants’ learning.

Some publications (Roeser, 2012; Sunderland, 2014; Sunderland et al., 2013, 2014), highlighted the role of role-playing games, problem-based learning, reflection on cases, student co-creation of curricular resources, and ethics research by students. They also noted that specific emotions such as joy, anxiety, shame and pride could arise through appraisals linked to learning as much as through ‘moral’ appraisals. Although such learning emotions were mentioned they were generally not theorized as linked to the learning process nor are they seen as academic emotions *per se*.

### Emotional Intelligence and Emotion Regulation

It was noted above that work on empathy has sometimes emphasized the risk of empathetic over-arousal leading to overwhelming distress (e.g. Berne, 2018; Hess et al., 2021). Likewise, the ethical insight perspective on moral emotions emphasizes that emotional information needs to be processed to contribute positively to moral judgement. Hence, the way in which emotional information is processed and used in cognition and social interaction– referred to as Emotional Intelligence (EI)– has been a focus of some publications in our review. Indeed the notion of empathy is particularly prominent in Goleman’s (1995) pop psychological account of EI. Hence it is not surprising that empathy was mentioned in several publications that also considered EI or social intelligence (Guntzburger et al., 2019; Hess & Fila, 2016; Lang et al., 2018; Lappalainen, 2015; Walther et al., 2017). Since empathy has been addressed above, our focus in this section is on the other ways in which EI and emotional self-regulation are used in studies of ethics in engineering education. In addition to recognition of emotion in others, competences seen as being part of EI include the ability to recognise emotions in oneself, to identify how emotions change and are modulated, to use emotions to facilitate thinking, and to regulate emotions in the self and others (this list of components is based on the Mayer and Salovey model of EI [Salovey et al., 2008]).

Guntzburger et al. (2019) found one student perceived EI to be a relevant competence to have as an engineer. Despite the weakness of this evidence, the authors concluded that EI is one among numerous approaches focusing on interpersonal skills that can support students’ learning in the context of ethical risk management. Lang et al. (2018) reported EI as an empirical outcome related to the effects of flipped classrooms on student perceptions of their professional and leadership skills. Lang et al. found that the flipped classroom may be more effective in promoting EI than the regular classroom. They report no association between EI and ethics learning. Taken together, this represents extremely weak evidence in relation to the role of EI in engineering ethics learning.

One component in EI is the capacity to control the type and intensity of experienced emotions, known as *emotion regulation* (Gross, 1999, 2014). Five of the included publications referred to emotional/self-regulation in the context of ethics in engineering education (Hess et al., 2021; Hess & Fila, 2016; Hess et al., 2017; Lappalainen, 2015; Walther et al., 2017). One empirical study by Hess et al. (2017) reported that an instructional intervention– designed to enhance students’ ability to recognise ethical issues– had no effect on developing students’ emotion regulation skills. Another empirical study (Hess et al., 2021) only mentioned emotional regulation as part of a conceptual discussion, reporting no associated empirical outcomes. There is, therefore, limited empirical evidence concerning emotion regulation in engineering ethics education in the papers in our review.

## Discussion

In this configurative review, our aim was to identify key themes, concepts, and theories currently in use in Engineering Ethics Education and to identify gaps in research topics, theories, and approaches used, with the purpose of critically evaluating the existing base of knowledge.

### Engagement across Theoretical Frameworks

We identified three major theoretical frameworks which underpin the theories used in most of the work on emotions in engineering ethics education: (A) Empathy, (B) Wider moral emotions, and (C) Emotional intelligence (EI)/emotion regulation.

These frameworks can easily be related to each other in a coherent way: empathy is conceptually linked to the moral emotions of distress-at-another’s-distress and compassion, and is also linked to the EI sub-component of recognising the emotion of others. Similarly, it is the risk of empathetic over-arousal and the need to process ethical insights from emotion that suggests the need for emotion regulation which is a component of EI. However almost all publications in our dataset treat only one of the three frameworks. As such they appear as distinct and their interrelationships are not explored. For example, authors who work on empathy (e.g. Hess, Strobel, Walther and their colleagues) typically do not refer to wider moral emotions. Indeed, the fragmentation can even be seen within some of the major theoretical groupings; among publications referring to wider moral emotions, for example, we found a separation between publications referring to moral emotions as sources of insight (Guntzburger, Kastenberg, Roeser, Sunderland) and those referring to moral emotions as providing intuitive judgements (Gelfand, Han, Kim and Jesiek). Only one paper sought to engage actively with both perspectives and to interrogate or test their claims (Lee et al., 2017). Similarly, publications typically dealt with one conceptualization of EI (typically Goleman’s pop-psychological model) while ignoring other (more research-based) conceptualizations (e.g. Mayer and Salovey’s). Few publications address the breadth of concepts and authors that make up the field. It seems likely that the field could be strengthened by a well-developed and integrated theoretical account of how empathy, other moral emotions, and emotion regulation are linked.

### Stronger Engagement with Theories of Emotion

The “emotional revolution” in psychology and education is now more than two decades old and there is a large and growing literature on emotions in education (Pekrun & Linnenbrink-Garcia, 2014). This includes a focus on achievement emotions (such as anxiety, pride, and shame– typically linked to measures of learning), topic emotions (such as love or anxiety– linked to the subject matter), social emotions (linked to appraisal of social relationships; c.f., Tormey, R. (2021)), and epistemic emotions (such as frustration– linked to a lack of understanding, or excitement– linked to the recognition of new ideas). Emotions are not simply a source of ethical motivation or information; they are inherent to the learning process. Remarkably, however, there was little engagement with theories of emotion in relation to education. For example, only Kim and Jesiek (2019) referenced academic emotions theory. We suggest, therefore, scholars should consider how ‘moral’ emotions may also be considered as ‘academic’ emotions that may arise in the engineering ethics *learning* process. The papers reviewed in this study largely focused on emotions and emotional skills as being learning goals, rather than as part of the learning process. To develop the field, it could be beneficial to theorize learning *with* emotion as well as learning *about* emotion in engineering ethics education.

The wider literature on emotion in moral decision making highlights that emotions may have disruptive as well as supportive effects on moral decision making. Anger, for example, has been linked to ethical action (e.g. Stanley et al., 2021), but also to disruption of decision making (Thiel et al., 2011). Similarly, compassion has been linked with both pro-social action and with some social bias (see Kotluk & Tormey, 2023, 2024) while empathic overarousal leading to withdrawal from the suffering of others has also been identified as a challenge for ethical action (Hoffman, 2008). Although this all suggests that emotion regulation should be an important component in engineering ethics education, there is little in our dataset to support the contention that emotion regulation or emotional intelligence play an important role in engineering ethics education practice today. Hence, this area could also benefit from further work.

Finally, engagement with theory can inform pedagogical choices. While our review found case study was the primary means for teaching engineering ethics (and to varying degrees engaging with emotion), arts-based approaches more broadly can also engage emotion and engineering ethics. Literary texts, such as *Frankenstein* (Shelly, 1818/ 2018/1818/ 2018; see for example treatment in Mawasi et al., 2022; Tansey, 2018), and film (Hitt & Lennerfors, 2022) are powerful tools for teaching engineering ethics because stories in texts and films elicit emotions and thus render them accessible for classroom discussions.

### Enhancing the Social in Conceptions of Emotion

As noted above, sociological perspectives on emotion in learning are found in reviews on emotions in education in other disciplines. Some sociologists understand emotions as complex “intersections of language, desire, power, bodies, social structures, subjectivity, materiality and trauma” (Zembylas, 2016, p. 546). These frameworks have allowed feminist and critical scholars to explore how social structures constrain and construct interpretations, expressions, and arousal of emotion (Turner & Stets, 2005), and they have been used to explore how different conceptualizations of emotions may reproduce or resist power structures and social inequalities (Ahmed, 2014; Boler, 1999; Zembylas, 2007). However, these types of socio-cultural perspectives on emotions were largely lacking in the publications included in our review. This seems to parallel the way in which micro-ethical perspectives appear to dominate over macro-ethical perspectives in engineering ethics education more generally, perhaps particularly in the US (see Polmear et al., 2019) where most of the publications in our dataset originated. We believe that the field could benefit greatly by engaging more social theorisations of emotion as they would allow researchers to explore questions of power, organisation, discrimination, and identity– questions which are at the heart both of emotion-in-engineering-ethics and emotion-in-education. Furthermore, employing research methods associated with such theorizations could open up new opportunities for empirical research in a field which is, at present, dominated by publications that are not based on systematic quantitative or qualitative data (Berne, 2018; Campbell et al; Campbell, 2013; Cruz et al., 2019; Davis, 2015; Frey, 2010; Gelfand, 2016; Han, 2015; Hess & Fila, 2016; Kastenberg, 2014; Lappalainen, 2015; Roeser, 2012; Sunderland, 2014; Sunderland et al., 2013, 2014; Walther et al., 2017).

The field can also benefit from Science, Technology, and Society (STS) perspectives to address a possible under-historicization of the connection between emotion and engineering ethics education. Although the sociotechnical systems perspective of STS lends itself well to understanding the complexity of engineering practice (Johnson & Wetmore, 2008), there has long been a division between STS and engineering ethics, which has been attributed to the activist versus scholar sub-cultures in STS (Herkert, 2006) and a hesitance to take normative stances, a practice that is more common in ethics research (Johnson & Wetmore, 2008). However, the historical and systems perspective of STS can help mitigate the enduring response in engineering to technological (and ethical) disasters of “we will recover and this will happen again” (Knowles, 2014, p. 229). Risk, as an example, is interconnected with emotions through risk appraisal and ethical decision making and is at the centre of disaster-STS, a subfield that accounts for historical context in understanding the impact of technology and engineering (Knowles, 2014). Another STS dimension that can expand the theorization of emotion in engineering ethics education is the *technological sublime*, an emotion of awe inspired by technology (Nye, 1996). In Nye’s work, for example, historical accounts of the sublime help explain the perception of technology and engineering in American culture that elicits both wonder and fear. The sublime thus presents another way to link the micro and macro processes in engineering ethics through emotion. Although there was limited engagement with such STS perspectives in the current corpus of work, this perhaps represents an opportunity for further development in the field of emotion in engineering ethics education.

### Limitations

It should be noted that research on emotion in engineering ethics education is a fast developing field. Our search process meant that our dataset only includes papers published before September 2020. New papers on emotion in engineering ethics education have certainly emerged since that time (however we know of no significant block of new papers that would fundamentally change the conclusions drawn here). A second limitation is that our search string focused on ‘ethics’ but did not include synonyms (such as ‘moral’) or related terms (such as ‘STS’). As we noted in the previous paragraph, the historic division between STS and ethics scholar sub-cultures means that we may have missed literature on the way emotion is integrated in STS education. Greater engagement with STS scholarship may be one way of bringing more social accounts of emotion into engineering ethics education.

## Conclusion

The last thirty years have seen a notable growth in research on emotions in education (Pekrun & Linnenbrink-Garcia, 2014), ethics (Haidt, 2003; Roeser, 2012), and engineering education (Lönngren et al., 2021, 2023, 2024). We expect that such growth will also take place in research at the intersection of those three fields: emotion in engineering ethics education. Our review shows that, so far, however, emotion is not broadly theorized in engineering ethics education publications. The results from our review indicate that researchers are typically working within very narrowly defined theoretical frameworks which address emotion-related concepts (such as empathy or emotional intelligence) but typically do so without theorizing emotion. A deeper engagement with theories of emotion may help authors to see the currently unexplored relationships between these emotion-related concepts. This, in turn, could lead to more substantial research questions and more consequential findings. We hope that this paper, by mapping the theories currently used, those ignored, and the spaces in-between, will contribute to developing a stronger field of research on emotion in engineering ethics education.

## Appendix I - Analytic Codes for in-Depth Analysis

The following analytic codes were derived inductively from a review of the original database of 17 publications from the systematic review database. Additional codes were added during the subsequent abductive coding of the 30 items retained for the final analysis.

- *Disciplinary grounding:* including cognitive neuroscience, sociology, philosophy, psychology, etc.
- *Central concepts, theories, frameworks, or models related to emotions*: including emotion words, empathy, moral emotions, cognitive views of emotion, emotion as a reason for action, among others.
- *Central concepts, theories, frameworks, or models related to ethics:* including ethical becoming, awareness, decision-making, risk, moral reasoning, moral judgement, ethical theories, ethical dilemmas, among others.
- *Central concepts, theories, frameworks, or models related to education:* including competencies, active, constructivist, cognitivist learning, student engagement, phenomenology informed pedagogy, among others.
- *Central concepts, theories, frameworks, or models related to engineering:* including engineering leadership, peace engineering, technology as a tool for good and bad, risky technology, among others.
- *Other central concepts, theories, frameworks, or models:* including discrimination, gender differences, adaptive complex systems, mindfulness, among others.
- *Pedagogical approaches:* including case studies, PBL, role play, written reflections, codes of ethics, service learning, among others.
- *Theorization status for emotion, or ethics, or education:* characterized as thoroughly theorized, shallowly theorized, or not theorized
- *Methods or methodology:* including linguistic analysis, statistical analysis, survey, mixed methods, group interviews, written reflections, pedagogical interventions, among others
- *Sample*: characterized as university students (undergraduate or graduate), professional engineers, or members of Engineers without borders
- *Educational context:* characterized as part of “normal” curriculum, or as elective course
- *Status of the paper:* either preliminary results or unclear description of results
- *Argument*: including need for more ethics training, need for analysis method, suggest a type of pedagogical intervention or teaching approach, provide good examples, engineering students should be taught that emotions are part of engineering, emotions are important for ethics, and emotive competence is important in industry
- *Focus on emotions:* including incidental (unexpected result), planned at outset, inherited from a larger project, but not focus of the described study

## Appendix II– 30 Publications included for Final Analysis

**Table:**

Paper #	Author (s)	Paper/Chapter Title	Year	Publication Title
1	Berne, R.	Global vision, technological skills, and systems thinking are essential qualities for peace engineering. Compassion too?	2018	World Engineering Education Forum - Global Engineering Deans Council, WEEF-GEDC
2	Campbell, R.C.	Caring in engineering: How Can Engineering Students Learn to Care? How Can Engineering Faculty Teach to Care?	2013	Engineering Education for Social Justice
3	Corman S., Sassu R., Bucuta M., Morar S., Ungureanu A., Kifor C., & Patil, A.	Discrimination of Various Vulnerable Groups - Perception Among the Students of the Faculty of Engineering (“Lucian Blaga” University of Sibiu)	2015	Proceedings of the 3RD International Engineering and Technology Education Conference & 7TH Balkan Region Conference on Engineering and Business Education
4	Cruz L.F., Moreno W.A., & Howell J.	Ethical education in engineering: A pedagogical proposal based on cognitive neurosciences and adaptative complex systems	2019	Proceedings of the American Society for Engineering Education (ASEE) Annual Conference and Exposition
5	Davis, M.	Engineers, emotions, and ethics	2015	In: Contemporary Ethical Issues in Engineering
6	Frey, E.J.	Teaching Virtue: Pedagogical Implications of Moral Psychology	2010	Science and Engineering Ethics
7	Gelfand, S.D.	Using Insights from Applied Moral Psychology to Promote Ethical Behavior Among Engineering Students and Professional Engineers	2016	Science and Engineering Ethics
8	Guntzburger Y., Pauchant T., & Tanguy P.	Empowering Engineering Students in Ethical Risk Management: An Experimental Study	2019	Science and Engineering Ethics
9	Gupta A., Elby A., & Philip T.M.	How engineering students think about the roles and responsibilities of engineers with respect to broader social and global impact of engineering and technology	2015	Proceedings of the American Society for Engineering Education (ASEE) Annual Conference and Exposition
10	Han, H.	Virtue ethics, positive psychology, and a new model of science and engineering ethics education	2015	Science and Engineering Ethics
11	Hashemian, Golnaz, and Michael C. Loui.	Can Instruction in Engineering Ethics Change Students’ Feelings about Professional Responsibility?	2010	Science and Engineering Ethics
12	Hess J., Miller S., Higbee S., Fore G., & Wallace J.	Empathy and ethical becoming in biomedical engineering education: a mixed methods study of an animal tissue harvesting laboratory	2021	Australasian Journal of Engineering Education
13	Hess, J. L. & N. D. Fila	The Development and Growth of Empathy among Engineering Students	2016	Proceedings of the American Society for Engineering Education (ASEE) Annual Conference and Exposition
14	Hess, J.L., Strobel, J., & Brightman, A.O.	The Development of Empathic Perspective Taking in an Engineering Ethics Course	2017	Journal of Engineering Education
15	James J.O., Svihla V., Qiu C., & Riley C.	Using design challenges to develop empathy in first-year courses	2018	Proceedings of the American Society for Engineering Education (ASEE) Annual Conference and Exposition
16	Kastenberg, W. E.	Ethics as analysis and ethics as feelings: The interplay of cognition and emotion on ethics education in biology, engineering and medicine.	2014	Ethics in Biology, Engineering & Medicine: An International Journal
17	Kim D. & Jesiek B.K.	Work-in-progress: Emotion and intuition in engineering students’ ethical decision making and implications for engineering ethics education	2019	Proceedings of the American Society for Engineering Education (ASEE) Annual Conference and Exposition
18	Lang D.H., Handley M., & Erdman A.M.	Flipped classroom and emotional learning in an engineering leadership development course	2018	Proceedings of the American Society for Engineering Education (ASEE) Annual Conference and Exposition
19	Lappalainen P.	Widening the industrial competence base: Integrating ethics into engineering education	2015	In: Contemporary Ethical Issues in Engineering
20	Lee E., Grohman M., Gans N., Tacca M., & Brown M.	The Roles of Implicit Understanding of Engineering Ethics in Student Teams’ Discussion	2017	Science and Engineering Ethics
21	Lee, E. A., Grohman, M. G., Gans, N., Tacca, M., & Brown, M. J.	Exploring implicit understanding of engineering ethics in student teams	2015	Proceedings of the American Society for Engineering Education (ASEE) Annual Conference and Exposition
22	Ramachandran M., Bairaktarova D., Woodcock A., & Bawareth O.M.	Differences in ethical decision making between experts and novices: A comparative study	2015	Proceedings of the American Society for Engineering Education (ASEE) Annual Conference and Exposition
23	Roeser, S.	Emotional Engineers: Toward Morally Responsible Design	2012	Science and Engineering Ethics
24	Strobel, J., Hess, J., Pan, R., Wachter Morris, C.A.	Empathy and care within engineering: Qualitative perspectives from engineering faculty and practicing engineers	2013	Engineering Studies
25	Sunderland M.E., Ahn J., Carson C., & Kastenberg W.E.	Making ethics explicit: Relocating ethics to the core of engineering education	2013	Proceedings of the American Society for Engineering Education (ASEE) Annual Conference and Exposition
26	Sunderland M.E., Taebi B., Carson C., & Kastenberg W.	Teaching global perspectives: engineering ethics across international and academic borders	2014	Journal of Responsible Innovation
27	Sunderland, M. E.	Taking Emotion Seriously: Meeting Students Where They Are.	2014	Science andEngineering Ethics
28	Thiel, C.E.,Connelly, S., Harkrider, L., Devenport, L.D., Bagdasarov, Z., Johnson, J.F., Mumford, M.D.	Case-Based Knowledge and Ethics Education: Improving Learning and Transfer Through Emotionally Rich Cases	2011	Science and Engineering Ethics
29	Troesch V.	Teaching Engineering Ethics: A Phenomenological Approach	2015	IEEE Technology and Society Magazine
30	Walther, J., Miller, S.E., & Sochacka, N.W.	A Model of Empathy in Engineering as a Core Skill, Practice Orientation, and Professional Way of Being	2017	Journal of Engineering Education
